# Single-photon quantum regime of artificial radiation pressure on a surface acoustic wave resonator

**DOI:** 10.1038/s41467-020-14910-z

**Published:** 2020-03-17

**Authors:** Atsushi Noguchi, Rekishu Yamazaki, Yutaka Tabuchi, Yasunobu Nakamura

**Affiliations:** 1 Research Center for Advanced Science and Technology (RCAST), The University of Tokyo, Meguro-ku, Tokyo 153-8904 Japan; 2 PRESTO, Japan Science and Technology Agency, Kawaguchi-shi, Saitama 332-0012 Japan; 3 Komaba Institute for Science (KIS), The University of Tokyo, Meguro-ku, Tokyo 153-8902 Japan; 4 Center for Emergent Matter Science (CEMS), RIKEN, Wako-shi, Saitama 351-0198 Japan

**Keywords:** Quantum mechanics, Single photons and quantum effects

## Abstract

Electromagnetic fields carry momentum, which upon reflection on matter gives rise to the radiation pressure of photons. The radiation pressure has recently been utilized in cavity optomechanics for controlling mechanical motions of macroscopic objects at the quantum limit. However, because of the weakness of the interaction, attempts so far had to use a strong coherent drive to reach the quantum limit. Therefore, the single-photon quantum regime, where even the presence of a totally off-resonant single photon alters the quantum state of the mechanical mode significantly, is one of the next milestones in cavity optomechanics. Here we demonstrate an artificial realization of the radiation pressure of microwave photons acting on phonons in a surface acoustic wave resonator. The order-of-magnitude enhancement of the interaction strength originates in the well-tailored, strong, second-order nonlinearity of a superconducting Josephson junction circuit. The synthetic radiation pressure interaction adds a key element to the quantum optomechanical toolbox and can be applied to quantum information interfaces between electromagnetic and mechanical degrees of freedom.

## Abstract (web summary)

The radiation pressure of light on a mechanical oscillator can be used to manipulate mechanical degrees of freedom in the quantum regime. Noguchi et al. use Josephson junctions to realize an artificial system where the radiation pressure of a single photon is stronger than the effect of dissipation.

## Introduction

The radiation pressure of electromagnetic field^1^ is one of the fundamental concepts in cavity optomechanics^2^. Even though the interaction is rather weak at the single-photon level, one can apply a strong drive field to enhance the effective coupling strength to reach the quantum regime^3–8^. Based on this interaction, ground-state cooling and quantum state control of mechanical oscillators have been reported on suspended membranes^3–5^, phononic-crystal cavities^6–8^, micro-toroidal resonators^9^, and bulk oscillators^10^. For such experiments, however, strong drive fields often restrict quantum-limited functionalities of the optomechanical systems by introducing noise, heat and other dissipations.

The single-photon quantum regime is reached when the radiation pressure of a single photon is strong enough to overcome other dissipations in the system^2^, where the quantum state of the mechanical mode is coherently controlled by the quantum of the electromagnetic field. However, such a strong radiation pressure interaction has been elusive in optomechanical systems studied so far, while there are a few experiments approaching this regime^11,12^.

**Fig. 1:**
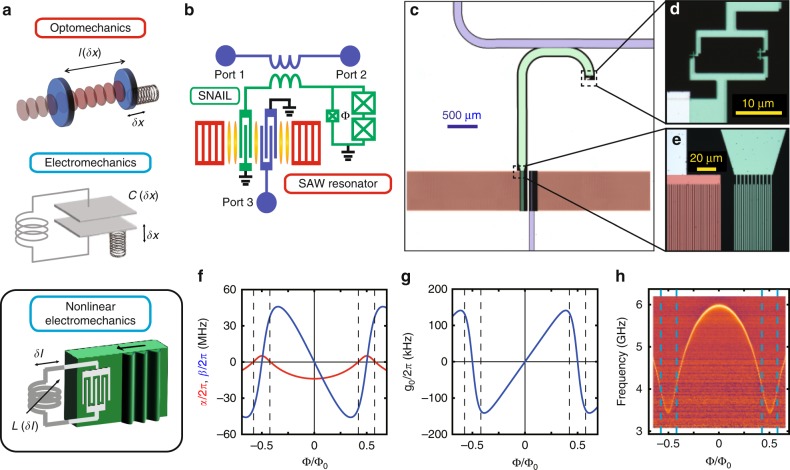
SAW–MW hybrid system for artificial radiation pressure interaction. **a** Variety of optomechanical systems. (top) Optomechanical system using an optical cavity. The effective cavity length *l* is modulated by the displacement of the mechanical oscillator *δx*. (middle) Electromechanics using a microwave lumped-element circuit. The capacitance *C* is modulated by the displacement of the mechanical oscillator. (bottom) Electromechanics with a nonlinear Josephson junction circuit and a SAW resonator. The inductance *L* is modulated by the current induced by transduction from acoustic waves. **b** Schematic of the SAW optomechanical system. A SAW resonator defined by Bragg mirrors (red) couples to a nonlinear MW resonator (green) via an interdigitated transducer. A SNAIL loop consisting of three Josephson junctions works as a nonlinear inductive element. Ports 1 and 2 are external feed lines for the MW resonator, and port 3 is that for the SAW resonator having a spatial mode shown in yellow. **c**–**e** False-colored micrographs of the sample. The colors of the electrodes correspond to the ones in the schematic in **b**. **d** Magnification of the SNAIL part. The three junctions form the SNAIL loop. **e** Zoom-up of a part of the SAW resonator and the interdigitated transducer. **f** Calculated nonlinearity of the MW resonator as a function of the magnetic flux Φ penetrating through the SNAIL loop. Red (blue) curve represents the self-Kerr (Pockels) nonlinearity *α* (*β*) of the MW resonator. **g** Calculated strength *g*_0_ of the artificial radiation pressure interaction induced by the nonlinearity of the SNAIL. **h** Spectrum of the nonlinear resonator as a function of Φ measured with a weak MW probe whose average intra-resonator photon number is much less than unity. Vertical dashed lines in **f**–**h** indicate flux bias conditions where the self-Kerr nonlinearity vanishes in the numerical simulation.

Here we introduce an artificial optomechanical system consisting of a surface acoustic wave (SAW) resonator and a superconducting circuit. The conventional radiation pressure arises from the frequency shift of the optical (or electrical) resonator depending on the displacement of the mechanical system (Fig. 1a). Instead, we utilize a superconducting circuit with Josephson junctions, which are known to be a versatile platform for engineering strong nonlinearity with negligible dissipation^13–16^. The current induced by the acoustic waves in a piezoelectric material modulates the inductive energy of the Josephson circuit, which results in the motion-dependent frequency shift of the electrical resonator. The enhanced artificial radiation pressure enables us to reach the single-photon quantum regime.

## Results

### System and the model

Our hybrid system is composed of a Fabry–Pérot-type SAW resonator defined by a pair of Bragg mirrors^17^, and a nonlinear microwave (MW) resonator (Fig. 1). They are coupled to each other via an interdigitated transducer (IDT) through the piezoelectric interaction. All the structures are made of aluminum evaporated on a ST-X quartz substrate (see the details in the “Method” section and Supplementary Note 1).

The nonlinear MW resonator consists of a short coplanar waveguide connected to the IDT on one end. On the other end it is grounded via a loop interrupted by one small and two large Josephson junctions with the Josephson energies $E^{\prime}$ and *E*_J_, respectively. The circuit element is called the Superconducting Nonlinear Asymmetric Inductive eLement (SNAIL)^18^. The SNAIL has the inductive energy

1 $$U(\theta )=-{E}_{{\rm{J}}}^{\prime}\cos \theta -2{E}_{{\rm{J}}}\cos \left(\frac{\phi -\theta }{2}\right),$$

where *θ* is the superconducting phase across the small junction, *ϕ* = 2*π*Φ/Φ_0_ is the reduced magnetic flux, Φ is the flux threading the loop, and Φ_0_ = *h*/2*e* is the flux quantum. The SNAIL capacitively shunted with the coplanar waveguide forms the nonlinear MW resonator, whose Hamiltonian reads (with *ℏ* = 1)

2 $${\hat{H}}_{{\rm{m}}}={\omega }_{{\rm{m}}}{\hat{a}}^{\dagger }\hat{a}+{\alpha }_{0}{\hat{a}}^{\dagger }{\hat{a}}^{\dagger }\hat{a}\hat{a}+\beta ({\hat{a}}^{\dagger }{\hat{a}}^{\dagger }\hat{a}+{\rm{h.c.}}).$$

Here $\hat{a}$ (${\hat{a}}^{\dagger }$) is the annihilation (creation) operator of a photon in the MW resonator, and *ω*_m_ is the resonance frequency. The terms with coefficients *α*_0_ and *β* represent the nonlinearities of the resonator corresponding to the self-Kerr and Pockels effects, respectively (see the details in Supplementary Note 2).

The Hamiltonian of the hybrid system consisting of the MW and SAW resonators is written as

3 $$\hat{H}={\hat{H}}_{0}+\hat{V},$$

where

4 $${\hat{H}}_{0}={\omega }_{{\rm{m}}}{\hat{a}}^{\dagger }\hat{a}+{\omega }_{{\rm{s}}}{\hat{b}}^{\dagger }\hat{b},$$

and

5 $$\hat{V}={\alpha }_{0}{\hat{a}}^{\dagger }{\hat{a}}^{\dagger }\hat{a}\hat{a}+\beta ({\hat{a}}^{\dagger }{\hat{a}}^{\dagger }\hat{a}+{\rm{h.c.}})+{g}_{{\rm{p}}}({\hat{a}}^{\dagger }\hat{b}+\hat{a}{\hat{b}}^{\dagger }).$$

Here $$\hat{b}$$$$({\hat{b}}^{\dagger })$$ is the annihilation (creation) operator of a phonon in the SAW resonator, *ω*_s_ is the resonance frequency, and *g*_p_ is the piezoelectric coupling strength between the SAW and MW resonators. By treating $$\hat{V}$$ as a perturbation (see the details in Supplementary Note 3), we obtain an effective interaction Hamiltonian

6 $${\hat{V}}_{{\rm{eff}}} 	=\left({\alpha }_{0}-\frac{3{\beta }^{2}}{{\omega }_{{\rm{m}}}}\right){\hat{a}}^{\dagger }{\hat{a}}^{\dagger }\hat{a}\hat{a}-\left(\frac{2{g}_{{\rm{p}}}\beta }{{\omega }_{{\rm{m}}}-{\omega }_{{\rm{s}}}}\right){\hat{a}}^{\dagger }\hat{a}({\hat{b}}^{\dagger }+\hat{b}),\\ 	 \equiv \alpha {\hat{a}}^{\dagger }{\hat{a}}^{\dagger }\hat{a}\hat{a}+{g}_{0}{\hat{a}}^{\dagger }\hat{a}({\hat{b}}^{\dagger }+\hat{b}),$$

under the rotating-wave approximation. This derivation is valid when {*ω*_m_, *ω*_s_, *ω*_m_ − *ω*_s_} ≫ {∣*α*_0_∣, ∣*β*∣, ∣*g*_p_∣} and *ω*_m_ ≫ *ω*_s_ are satisfied. The second term on the right-hand side represents an artificial radiation pressure interaction analogous to the Pockels effect. On the other hand, the undesired first term corresponds to a self-Kerr nonlinearity, which can be eliminated by finding experimental conditions where *α* vanishes. As we will show later, this condition mitigates the saturation effect and provides full functionality of the realized artificial radiation pressure.

Figure 1f shows the calculated strengths of the self-Kerr nonlinearity *α* and Pockels nonlinearity *β* for the parameters of our sample. Notably, *α* vanishes at certain flux bias conditions Φ = {Φ_*α*=0_, Φ_0_−Φ_*α*=0_} mod Φ_0_ (vertical dashed lines in Fig. 1f–h), while *β* remains finite at the conditions. In canonical optomechanical systems, the resonance frequency of the optical (or electrical) resonator is directly affected by the displacement of the mechanical oscillator. Here, in contrast, the resonance frequency of the MW resonator is modulated by the current excited by the mechanical oscillations through the piezoelectric effect, resulting in the Pockels nonlinearity and the synthetic optomechanical coupling. Figure 1g shows the calculated strength *g*_0_ of the artificial radiation pressure interaction. It is of importance that *g*_0_ takes a large value at the flux bias condition where *α* vanishes.

### Nonlinearity of the circuit

**Fig. 2:**
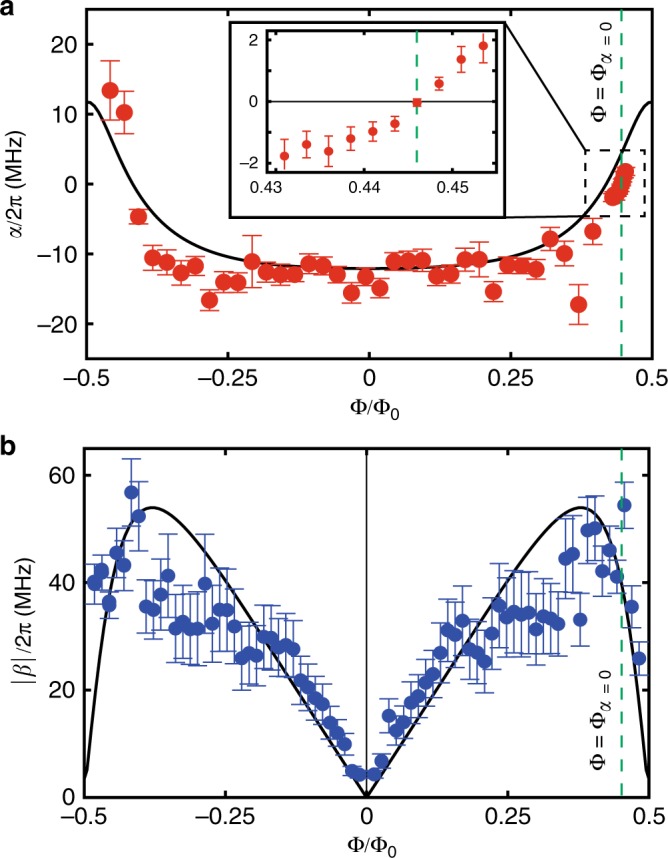
Nonlinearity of the MW resonator. **a** Self-Kerr nonlinearity *α* as a function of the flux through the SNAIL loop. Inset shows an enlarged view around Φ = Φ_*α*=0_. **b** Absolute value of the Pockels nonlinearity ∣*β*∣, obtained from the phonon-to-photon conversion experiment, as a function of the magnetic flux bias. The curve in each panel shows the result of the numerical simulation without any fitting parameters. Green dashed lines indicate Φ = 0.445Φ_0_ ≡ Φ_*α*=0_. The theoretical curves are the same as those in Fig. 1f. Error bars represent standard errors.

Figure 2a shows the experimentally determined self-Kerr nonlinearity *α* as a function of the flux bias. For that, we measure the shift of the MW resonator frequency per average intra-resonator probe photon number as a function of the flux bias. The observed self-Kerr nonlinearity changes its sign near Φ = ±0.5Φ_0_, as expected. The relatively large scattering of the experimental data points are presumably due to the uncertainty in the determination of the probe photon number in the resonator because of the strongly flux-dependent loss rates of the MW resonator (see Supplementary Figure 1). As shown in the inset, *α* vanishes at Φ_*α*=0_ ≡ 0.445Φ_0_. At this bias point, the MW resonator frequency is *ω*_m_/2*π* = 3.85 GHz (Fig. 1h), largely detuned from the SAW resonator frequency *ω*_s_/2*π* = 785.25 MHz.

To evaluate the strength of Pockels nonlinearity *β*, we perform a phonon-to-photon conversion experiment from the SAW resonator to the MW resonator. We irradiate the MW resonator with the red-sideband drive at frequency *ω*_d_ ~ *ω*_m_ − *ω*_s_. Under the rotating wave approximation in the resolved-sideband limit, i.e., *κ* ≪ *ω*_s_, where *κ* is the total decay of the MW resonator, the Hamiltonian becomes

7 $$\hat{H}=\Delta {\hat{a}}^{\dagger }\hat{a}+{g}_{0}\sqrt{{n}_{{\rm{d}}}}({\hat{a}}^{\dagger }\hat{b}+\hat{a}{\hat{b}}^{\dagger }),$$

where *n*_d_ is the average photon number of the drive field in the MW resonator and Δ ≡ *ω*_m_ − *ω*_d_ − *ω*_s_ is the detuning. The details of the derivation are presented in Supplementary Note 4. When the drive field is tuned to the red-sideband transition, i.e., Δ = 0, the two resonators are parametrically coupled to each other. Then, the excitation of the SAW resonator is converted to the excitation of the MW resonator, and its output MW power *P*_out_ can be written as

8 $${P}_{{\rm{out}}}=\hslash {\omega }_{{\rm{m}}}{\kappa }_{{\rm{ex}}}{n}_{{\rm{s}}}\frac{4{C}_{0}{n}_{{\rm{d}}}}{{(1+{C}_{0}{n}_{{\rm{d}}})}^{2}},$$

where ${C}_{0}\equiv 4{g}_{0}^{2}/(\kappa \Gamma )$ is the single-photon cooperativity between the SAW and MW resonators, *κ* and Γ are the respective total loss rates, and *κ*_ex_ is the external coupling of the MW resonator. The average intra-resonator photon number *n*_d_ of the drive field and the intra-resonator phonon number *n*_s_ of the SAW resonator are calibrated by the saturation effect and the Stark shift of the MW resonator, respectively (see Supplementary Figs. 4 and 5). Here, for the phonon-to-photon conversion, we use a weak drive field which provides a small *n*_d_ (~0.01) to avoid saturating the MW resonator. Therefore, we can evaluate *C*_0_ from Eq. (8). Acccording to the definition of *g*_0_ in Eq. (6), the relation between *C*_0_ and *β* follows:

9 $${C}_{0}=\frac{16{g}_{{\rm{p}}}^{2}{\beta }^{2}}{\kappa \Gamma {({\omega }_{{\rm{m}}}-{\omega }_{{\rm{s}}})}^{2}},$$

from which we evaluate the strength of the Pockels nonlinearity $\beta \,[=(\sqrt{{C}_{0}\kappa \Gamma }/4g)({\omega }_{{\rm{m}}}-{\omega }_{{\rm{s}}})]$ shown in Fig. 2b. The overall behavior agrees well with the theoretical prediction.

### Single photon quantum regime

**Fig. 3:**
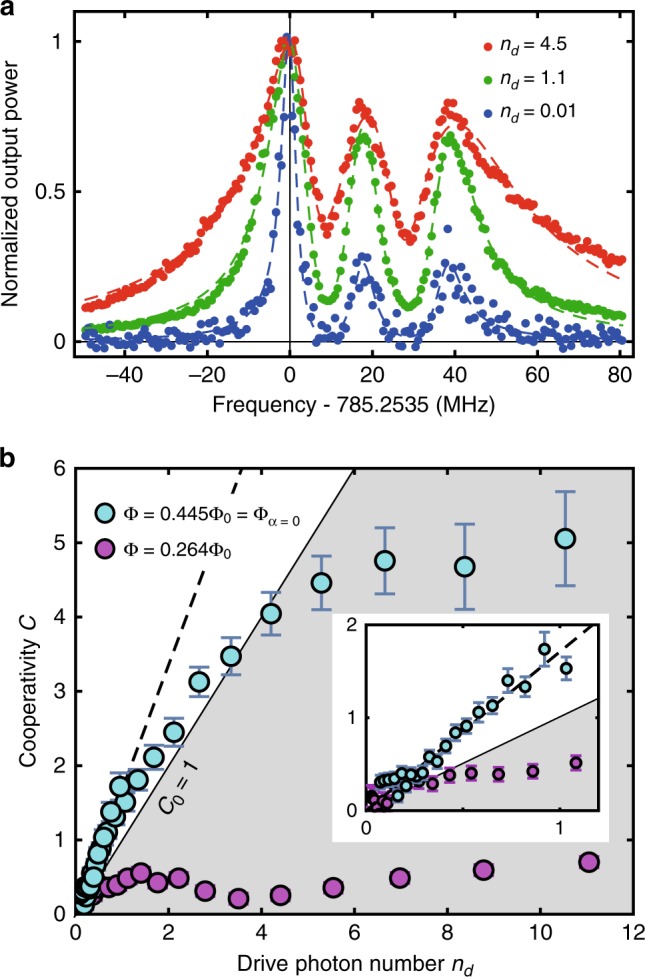
Strong artificial radiation pressure. **a** Normalized output power of the MW signal coherently up-converted from the SAW excitations as a function of the detuning of the SAW drive frequency (dots). Three datasets are for different drive powers represented by the average number, *n*_d_, of drive photons in the MW resonator. Dashed curves are the results of fittings with a sum of three Lorentzians in the complex plane. The left-most peak is from the fundamental transverse mode of the SAW resonator, while the two other peaks are due to higher-index transverse modes. **b** Cooperativity *C* as a function of the drive photon number *n*_d_. White area corresponds to the single-photon quantum regime, i.e., *C*_0_ > 1. Cyan dots are the experimental data at Φ = 0.445Φ_0_ = Φ_*α*=0_. Purple dots are taken at Φ = 0.264Φ_0_, where *α* ≠ 0 and the saturation takes place at lower power. Black dashed line is the linear fit for the cyan dots in the low-power region. Inset shows an enlarged view near the origin. Error bars represent standard errors.

We apply a stronger red-sideband drive to obtain a larger cooperativity with the artificial radiation pressure. This results in the increase of the effective decay rate of the SAW resonator through the optomechanical damping. Figure 3a shows the spectra of the SAW resonator in the presence of the optomechanical damping rate Γ_opt_. The total linewidth Γ_all_ of the spectrum is given by

10 $${\Gamma }_{{\rm{all}}}=\Gamma +{\Gamma }_{{\rm{opt}}}=(1+C)\Gamma ,$$

from which we evaluate the cooperativity *C*.

Figure 3b shows the cooperativity *C* as a function of the drive photon number *n*_d_ at Φ = 0.445Φ_0_ = Φ_*α*=0_ and Φ = 0.264Φ_0_, respectively. Because of the absence of the self-Kerr nonlinearity, the saturation effect is much less pronounced at Φ = Φ_*α*=0_, allowing us to drive the system, as in other conventional optomechanical systems, to enhance the radiation pressure interaction. The cooperativity reaches 5 at high drive power. The remaining saturation effect is presumably due to the higher-order nonlinearities beyond the third order. The slope of the cooperativity for the small drive photon number corresponds to the single-photon cooperativity *C*_0_, which is determined to be 1.7 ± 0.1 from the linear fit in Fig. 3. Thus, the single-photon quantum regime *C*_0_ > 1 is achieved here. From this value, the optomechanical coupling strength is evaluated to be *g*_0_/2*π* = 190 kHz, which agrees well with the calculation shown in Fig. 1g. It is also consistent with the estimation from the peak power of the up-conversion signal [Eq. (9)], which gives *g*_0_/2*π* = 230 kHz.

## Discussions

**Fig. 4:**
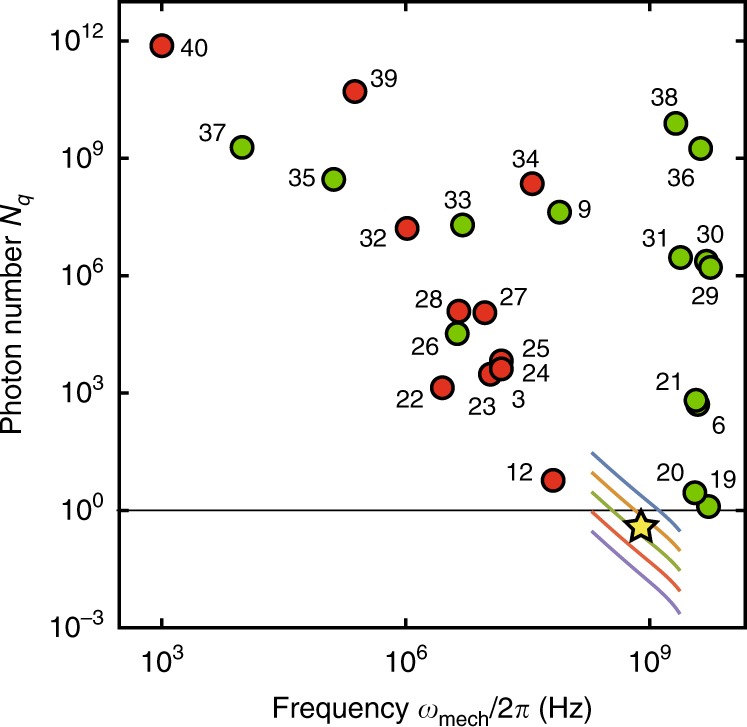
Single-photon quantum regime. Each data point shows intra-resonator drive photon number *N*_q_ necessary for the quantum-limited quadrature measurement of phonons as a function of mechanical eigenmode frequency *ω*_mech_∕2*π*. Green (red) circles indicate the values found in other opto-(electro-)mechanical systems with radiation pressure interaction. The yellow star shows the value obtained in this work. Color curves show the expectations from numerical simulations. Different colors correspond to the values of *E*_J_∕*E*_C_ = {10^5^, 10^4^, 10^3^, 10^2^, 10} from top to bottom. A list of the references indicated by the numbers next to the circles is presented in the reference list.

Having established the single-photon quantum regime, we compare various realizations of optomechanical systems^3,6,9,12,19–40^ from the viewpoint of quantum-limited measurement of phonons. Figure 4 shows the minimum intra-resonator photon number *N*_q_ = (*n*_th_ + 1/2)/*C*_0_, whose back-action shot noise on the mechanical mode becomes dominant over the thermal and vacuum noises^2^. Here ${n}_{{\rm{th}}}=1/({{\mathrm{{e}}}}^{\hslash {\omega }_{{\rm{mech}}}/{k}_{{\rm{B}}}T}-1)$, *ω*_mech_ is the mechanical mode frequency, *T* is the bath temperature, and *k*_B_ is Boltzmann constant. The experiment was conducted at *T* = 40 mK, which gives *N*_q_ of 0.67 ± 0.04. *N*_q_ becomes less than unity when the kick by a single intra-resonator photon is larger than the mechanical oscillator beyond its noise amplitude of the motion. In this regime, the quantum-limited quadrature measurement of the mechanical oscillator and the mechanically induced transparency of the MW resonator can be realized with single intra-resonator photons. The current device is in the resolved-sideband limit, however, such that the incident drive is strongly filtered by the resonator and needs to be a large amplitude even if the required intra-resonator photon number is less than unity. To solve the problem, we can use a triple resonance technique^41^ to improve the coupling efficiency of the incident drive and utilize a quantum feature of the drive field.

The single-photon quantum regime is also useful for the quantum control of the mechanical oscillator through the radiation pressure interaction. When *N*_q_ is less than unity, we can safely use a two-level system, i.e., a qubit, instead of a nearly harmonic microwave resonator. Then, quantum control of the SAW resonator can be readily demonstrated. Recently, quanta of acoustic waves are being controlled and monitored by using a resonantly^42,43^ or dispersively^44,45^ coupled superconducting qubit. In contrast to those demonstrations, the radiation pressure interaction between a SAW resonator and a superconducting qubit gives rise to a ‘spin’ dependent force, which can be used, e.g., for generating a Schödinger’s cat state of a SAW resonator, as recently demonstrated in a vibrational mode in a trapped-ion experiment^46^. The spin-dependent force also enables fast entangling gates in analogy with those in trapped-ion systems^47,48^.

Moreover, as the numerical simulations indicate (color lines in Fig. 4), we can readily increase the coupling strength further by using a SAW resonator with higher frequency to reduce the detuning from the MW resonator. The condition *g*_0_/*κ* > 1 is also within the scope of the future experiment, where the presence of a single phonon shifts the resonance frequency of the MW resonator by more than its linewidth^49^. It will then allow for observation of quantum jumps between phonon Fock states^50^.

## Method

### Sample parameters

The parameters in the sample are the following. At zero flux bias, the resonance frequency of the MW resonator is *ω*_m_/2*π* = 5.98 GHz, and the internal and total loss rates are 10 and 55 MHz, respectively. At Φ = Φ_*α*=0_ ≡ 0.445Φ_0_, the resonance frequency is 3.85 GHz, the internal loss rate *κ*_in_/2*π* = 3 MHz, and the external loss rate *κ*_ex_/2*π* = 17 MHz. The resonance frequency of the SAW resonator is found to be *ω*_s_/2*π* = 785.25 MHz, together with the total loss rate Γ/2*π* = 4.4 kHz. The external coupling rate is designed to be Γ_ex_/2*π* = 0.6 kHz. The total loss rate of the MW resonator *κ*/2*π* = 20 MHz is much smaller than the resonance frequency of the SAW resonator. The piezoelectric coupling strength evaluated from the frequency shift of the SAW resonator under a strong drive is *g*_p_/2*π* = 6.4 MHz^17^. The strength of the self-Kerr nonlinearity at zero flux bias is determined as *α*_0_/2*π* = −13.0 MHz (see the details in Supplementary Note 5). The bath temperature in the experiment was at *T* = 40 mK.

## Supplementary information

Supplementary Information

Peer Review File

## Data Availability

The data that support the findings of this study are available from the corresponding author upon reasonable request.
