# Supplementary material for: Single-photon quantum regime of artificial radiation pressure on a surface acoustic wave resonator
Source: Nat Commun. 2020 Mar 17;11:1183. doi: 10.1038/s41467-020-14910-z (PMC7078202; doi:10.1038/s41467-020-14910-z)
Supplement: Supplementary file 1 — Supplementary Information [file 41467_2020_14910_MOESM1_ESM.pdf]

Supplementary information for Single-photon quantum regime of artificial radiation pressure on a surface acoustic wave resonator

Atsushi Noguchi[1,2,3],* Rekishu Yamazaki[1], Yutaka Tabuchi[1], and Yasunobu Nakamura[1,4]
[1]*Research Center for Advanced Science and Technology (RCAST), The University of Tokyo, Meguro-ku, Tokyo, 153-8904, Japan,*
[2]*PRESTO, Japan Science and Technology Agency, Kawaguchi-shi, Saitama 332-0012, Japan,*
[3]*Komaba Institute for Science (KIS), The University of Tokyo, Meguro-ku, Tokyo, 153-8902, Japan,*
[4]*Center for Emergent Matter Science (CEMS), RIKEN, Wako-shi, Saitama 351-0198, Japan*
(Dated: January 24, 2020)

SUPPLEMENTARY NOTE 1: SAMPLE

The circuit was fabricated on a 500-μm-thick ST-X cut quartz substrate. The Bragg mirrors, the interdigitated transducers (IDTs), the nonlinear microwave (MW) resonator, and the coplanar waveguides for the external feed lines were simultaneously patterned in a wet-etching process from a 50-nm-thick evaporated aluminum film. The Bragg mirrors have 750 fingers each. The IDT for the external coupling has a pair of four fingers, and the IDT connected to the MW resonator has a pair of ten fingers (Fig. 1e in the main text). All those fingers have a width and a spacing of 1 μm. The length of the surface-acoustic-wave (SAW) resonator, the inner distance between the Bragg mirrors, is 240 μm. The widths of the Bragg mirrors and the IDTs are 500 μm. The Josephson junctions for the SNAIL are made from Al/AlO$_x$/Al junctions, which are simultaneously fabricated by the shadow evaporation technique with the bridgeless resist mask. The size of the junctions are 150 $\times$ 150 nm for the small one and 300 $\times$ 300 nm for the large ones.

Supplementary Figure 1 shows the resonance frequency and the loss rates of the MW resonator as a function of the magnetic flux in the SNAIL loop. Note that the loss rates are periodically fluctuating depending on the flux bias. The periodic modulation is presumably caused by the resonant acoustic radiation from the MW resonator. The internal loss rate $\kappa_{\rm in}$ is divided into the electric loss $\kappa_{\rm e}$ and the acoustic radiation loss $\kappa_{\rm a}$ from the MW resonator. The part of the acoustic radiation is picked up by the IDT electrode of the SAW resonator, and thus $\kappa_{\rm a} = \kappa_{\rm cross} + \kappa_{\rm rad}$, where $\kappa_{\rm cross}$ is the external coupling rate of the MW resonator though acoustic waves to the SAW input port (port 3 in Fig. 1b) and $\kappa_{\rm rad}$ is the acoustic radiation rate to the environment. Supplementary Figure 1d shows the acoustic external coupling rate $\kappa_{\rm cross}$ of the MW resonator to the SAW input port (port 3 in Fig. 1b).

SUPPLEMENTARY NOTE 2: NONLINEAR RESONATOR WITH SNAIL

Our SNAIL has a single small junction and two large junctions. It is shunted with a large capacitor whose single-electron charging energy $E_{\rm C}$ is estimated to be $h \times 35$ MHz. To determine the Josephson energies in the device, we fit the flux-dependent spectrum in Fig. 1g and obtain $E'_{\rm J} = h \times 47.5$ GHz and $E_{\rm J} = h \times 163.5$ GHz, respectively. Supplementary Figure 2 shows the inductive energy $U(\theta)$ of the SNAIL, given by Eq.(1) in the main text, in units $E_{\rm J}$. For $\Phi \neq 0$, the parity symmetry is broken and the Pockels nonlinearity appears. The inductive energy is expanded around the minimum at θ_0 in a power series of $\tilde{\theta} \equiv \theta - \theta_0$ as

$$U(\tilde{\theta})/E_{\rm J} = -\sum \chi_i \tilde{\theta}^i. \tag{S1}$$

The Hamiltonian of this nonlinear resonator in the transmon limit ($E_{\rm J} \gg E_{\rm C}$) reads

$$\hat{H} = 4E_{\rm C}\hat{N}^2 - E_{\rm J}\sum \chi_i \hat{\theta}^i, \tag{S2}$$

where $\hat{N}$ is the number operator of the excess Cooper pairs in the superconducting electrode connected to the ground via the SNAIL. For the phase operator $\hat{\theta}$, we omit the tilde for simplicity. This Hamiltonian can be rewritten with

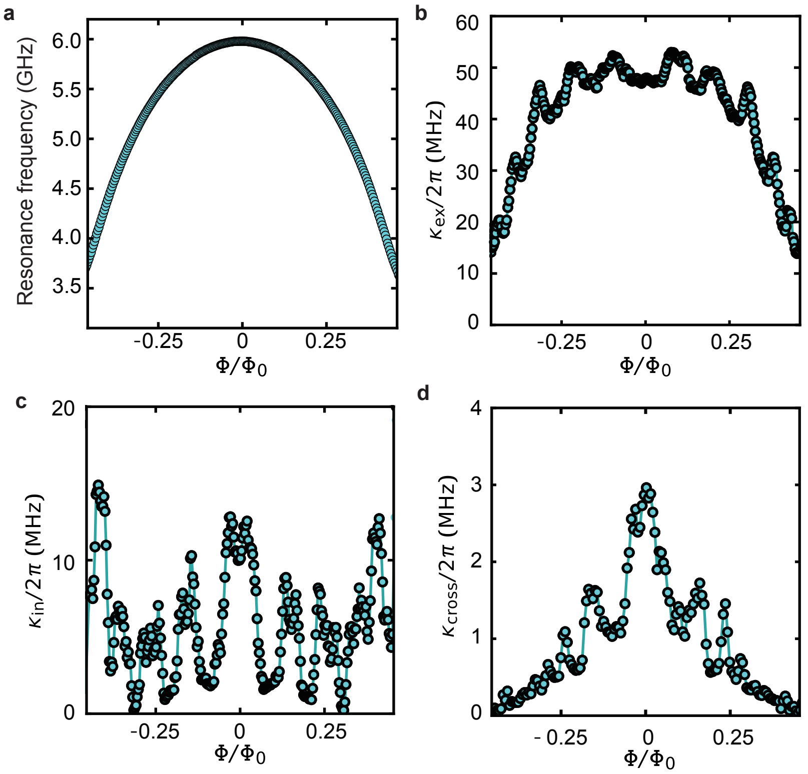

Supplementary Figure 1: Parameters of the nonlinear MW resonator as a function of the flux bias. **a.** The resonance frequency $\omega_{\mathrm{m}}/2\pi$, **b.** external loss rate κ_{ex}, **c.** internal loss rate κ_{in} and **d.** acoustic external loss rate to the IDT electrode are evaluated from the spectroscopy at the low-power limit with input-output theory.

the creation and annihilation operators as

$$\begin{aligned}\hat{H} &= \left(\sqrt{16E_{\mathrm{C}}E_{\mathrm{J}}\chi_2} - 12E_{\mathrm{C}}\frac{\chi_4}{\chi_2}\right)\hat{a}^\dagger\hat{a} \\ &\quad -3E_{\mathrm{C}}\left(\frac{\chi_2 E_{\mathrm{J}}}{E_{\mathrm{C}}}\right)^{1/4}\frac{\chi_3}{\chi_2}(\hat{a}^\dagger\hat{a}^\dagger\hat{a} + \mathrm{h.c.}) \\ &\quad -6E_{\mathrm{C}}\frac{\chi_4}{\chi_2}\hat{a}^\dagger\hat{a}^\dagger\hat{a}\hat{a} + O(\hat{a}^5), \end{aligned} \tag{S3}$$

where

$$\hat{a} = \left(\frac{E_{\mathrm{C}}}{h^2\chi_2 E_{\mathrm{J}}}\right)^{1/4}\left(i\hat{N} + \sqrt{\frac{\chi_2 E_{\mathrm{J}}}{4E_{\mathrm{C}}}}\hat{\theta}\right). \tag{S4}$$

The Pockels (second-order) and self-Kerr (third-order) nonlinearities appear in Eq.(S3). This relates the circuit parameters to the coefficients of the nonlinear tems in Eq. (2) of the main text as

$$\alpha_0 = -12E_{\mathrm{C}}\frac{\chi_4}{\chi_2}, \tag{S5}$$

$$\beta = -3E_{\mathrm{C}}\left(\frac{\chi_2 E_{\mathrm{J}}}{E_{\mathrm{C}}}\right)^{1/4}\frac{\chi_3}{\chi_2}. \tag{S6}$$

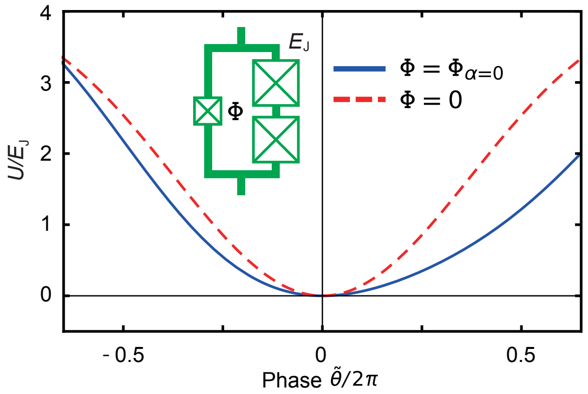

Supplementary Figure 2: Inductive energy $U(\tilde{\theta})$ of the SNAIL as a function of the phase difference $\tilde{\theta}$ across the small Josephson junction. The inset shows the circuit model of the SNAIL.

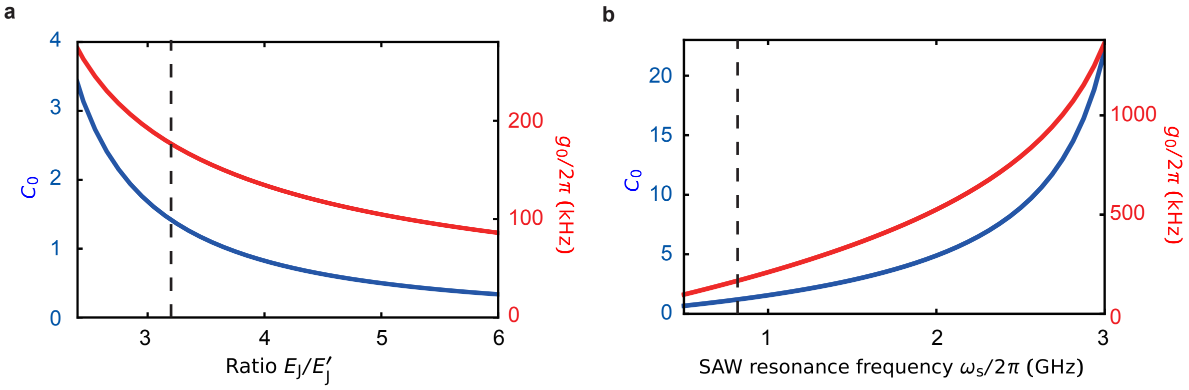

Supplementary Figure 3: Estimation of the optomechanical coupling strength. Blue and red curves show the single-photon cooperativity C_0 and the strength of the single-photon radiation pressure interaction g_0, respectively. The Josephson energy E_J of the larger junctions in the SNAIL and the SAW resonator frequency $\omega_s/2\pi$ are swept in **a** and **b**, respectively. Vertical dashed lines indicate the parameters in the current experiment. Other parameters are set to the values obtained in the experiment.

SUPPLEMENTARY NOTE 3: ARTIFICIAL OPTOMECHANICAL COUPLING

The total Hamiltonian of the hybrid system consisting of a nonlinear MW resonator and a SAW resonator piezoelectrically coupled to each other is described without rotating wave approximation as

$$\hat{H} = \hat{H}_0 + \hat{V}_0, \tag{S7}$$

where

$$\hat{H}_0 = \omega_m \hat{a}^\dagger \hat{a} + \omega_s \hat{b}^\dagger \hat{b}, \tag{S8}$$

and

$$\begin{aligned} \hat{V}_0 &= \beta(\hat{a}^\dagger \hat{a}^\dagger \hat{a} + \text{h.c.}) + \alpha_0 \hat{a}^\dagger \hat{a}^\dagger \hat{a}\hat{a}, \\ &\quad + g_p(\hat{a}^\dagger + \hat{a})(\hat{b}^\dagger + \hat{b}). \end{aligned} \tag{S9}$$

The parameters and the operators are defined in the main text. g_p is the piezoelectric coupling coefficient of the nonlinear MW resonator and SAW resonator.

By treating $\hat{V}_0$ as a perturbation, we find an effective Hamiltonian as

$$\begin{aligned}
\hat{H}_{\rm eff} &= \hat{H}_0 + \left(\alpha_0 - \frac{3\beta^2}{\omega_{\rm m}}\right)\hat{a}^\dagger\hat{a}^\dagger\hat{a}\hat{a} \\
&\quad - \left(\frac{g_{\rm p}\beta}{\delta} + \frac{g_{\rm p}\beta}{\omega_{\rm m}}\right)\hat{a}^\dagger\hat{a}(\hat{b}^\dagger + \hat{b}) \\
&\quad - \frac{g_{\rm p}\beta\omega_{\rm s}}{2\omega_{\rm m}\delta}(\hat{a}^\dagger\hat{a}^\dagger\hat{b} + {\rm h.c.}) \\
&\quad + \frac{g_{\rm p}\beta\omega_{\rm s}}{2\omega_{\rm m}(\omega_{\rm m}+\omega_{\rm s})}(\hat{a}^\dagger\hat{a}^\dagger\hat{b}^\dagger + {\rm h.c.}) \\
&\quad - \frac{2g_{\rm p}\alpha_0}{\omega_{\rm m}+\omega_2}(\hat{a}^\dagger\hat{a}\hat{a}\hat{b} + {\rm h.c.}) \\
&\quad - \frac{2g_{\rm p}\alpha_0}{\omega_{\rm m}-\omega_2}(\hat{a}^\dagger\hat{a}\hat{a}\hat{b}^\dagger + {\rm h.c.}) \\
&\quad - \frac{2\alpha_0\beta}{\omega_{\rm m}}(\hat{a}^\dagger\hat{a}^\dagger\hat{a}\hat{a}\hat{a} + {\rm h.c.}),
\end{aligned} \tag{S10}$$

where $\delta = \omega_{\rm m} - \omega_{\rm s}$ is the detuning between the MW and SAW resonators. This calculation is valid when $\{\omega_{\rm m},\ \omega_{\rm s}, \delta\} \gg \{|\alpha_0|,\ |\beta|,\ |g_{\rm p}|\}$ is satisfied. While the second term on the right-hand side gives the self-Kerr nonlinearity, the third term leads to the radiation pressure interaction, and the fourth term introduces dynamical Casimir effect. When $\alpha_0 = 3\beta^2/\omega_{\rm m}$, the self-Kerr nonlinearity vanishes, and the effective Hamiltonian is rewritten as

$$\hat{H}_{\rm eff} = \hat{H}_0 - 2\frac{g_{\rm p}\beta}{\delta}\hat{a}^\dagger\hat{a}(\hat{b}^\dagger + \hat{b}), \tag{S11}$$

$$= \hat{H}_0 + g_0\hat{a}^\dagger\hat{a}(\hat{b}^\dagger + \hat{b}), \tag{S12}$$

with the rotating wave approximation and a large detuning ($\delta \sim \omega_{\rm m}$).

SUPPLEMENTARY NOTE 4: LINEARIZED HAMILTONIAN

We irradiate the MW drive at frequency $\omega_{\rm d}$, the annihilation operator of the MW resonator becomes

$$\hat{a} \to e^{-i\omega_{\rm d}t}\Omega + \hat{a} \tag{S13}$$

and the interaction term becomes

$$\hat{V} = g_0\hat{a}^\dagger\hat{a}(\hat{b}^\dagger + \hat{b}) \tag{S14}$$

$$\begin{aligned}
&\to g_0(e^{-i\omega_{\rm d}t}\Omega\hat{a}^\dagger + e^{i\omega_{\rm d}t}\Omega^*\hat{a})(\hat{b}^\dagger + \hat{b}) \\
&\quad + g_0(|\Omega|^2 + \hat{a}^\dagger\hat{a})(\hat{b}^\dagger + \hat{b}),
\end{aligned} \tag{S15}$$

where Ω is the complex amplitude of the MW drive.

On the rotating frame with a unitary operator

$$\exp\left[-i(\omega_{\rm d}+\omega_{\rm s})t\hat{a}^\dagger\hat{a} - i\omega_{\rm s}t\hat{b}^\dagger\hat{b}\right], \tag{S16}$$

the Hamiltonian becomes

$$\begin{aligned}
\hat{H}_{\rm eff} = &\ (\omega_{\rm m} - \omega_{\rm d} - \omega_{\rm s})\hat{a}^\dagger\hat{a} \\
&+ g_0(\Omega\hat{a}^\dagger + \Omega^*\hat{a})(\hat{b}^\dagger + \hat{b}) \\
&+ g_0(|\Omega|^2 + \hat{a}^\dagger\hat{a})(e^{i\omega_{\rm s}t}\hat{b}^\dagger + e^{-i\omega_{\rm s}t}\hat{b}).
\end{aligned} \tag{S17}$$

When the bandwidth of the MW resonator κ and the strength of the radiation pressure interaction g_0 are both smaller than $\omega_{\rm s}$, we can apply the rotating approximation to eliminate the third term and obtain the linearized Hamiltonian.

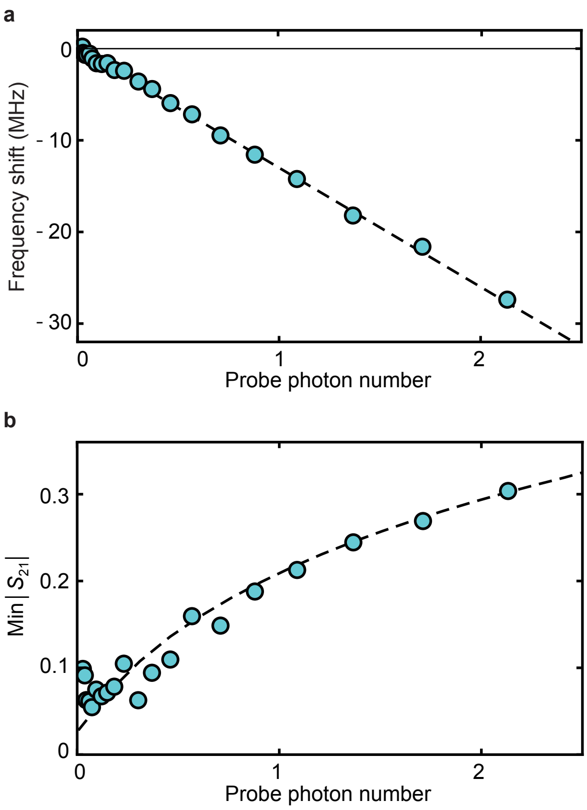

Supplementary Figure 4: Calibration of the resonator photon number and the nonlinearity of the MW resonator at zero flux bias. **a.** Frequency shift of the nonlinear MW resonator as a function of the probe power represented by the intra-resonator photon number. **b.** Saturation of the nonlinear MW resonator as a function of the probe power. The vertical axis shows the minimum values of the normalized transmission coefficient $|S_{21}|$ at the resonance of the MW resonator. The self-Kerr nonlinearity makes the MW resonator saturated.

SUPPLEMENTARY NOTE 5: SELF-KERR NONLINEARITY

We characterize the amount of the self-Kerr nonlinearity of the MW resonator by measuring the frequency shift as a function of the probe power. Supplementary Figures 4a and 4b show the frequency shift and the saturation of the absorption in the MW resonator at zero flux bias, respectively. To analyze the result, we solve the master equation of the resonator with the third-order nonlinearity and fit the experimental data. In the steady state, it fulfills

$$i[\hat{\rho}, \hat{H}_{\rm fit}] + \hat{L}[\hat{\rho}] = \dot{\hat{\rho}} = 0, \tag{S18}$$

where $\hat{\rho}$ is the density-matrix operator of the MW resonator, $\hat{L}$ is the Lindblad superoperator, and

$$\begin{aligned} \hat{H}_{\rm fit} &= \sqrt{4A_{\rm m}P_{\rm m}\kappa_{\rm ex}/\hbar\omega_{\rm m}}(\hat{a}^\dagger + \hat{a}) \\ &\quad + \Delta\hat{a}^\dagger\hat{a} + \alpha_0\hat{a}^\dagger\hat{a}^\dagger\hat{a}\hat{a}. \end{aligned} \tag{S19}$$

Here, $A_{\rm m}$ is the attenuation through the input line of the MW feedline, and $P_{\rm m}$ is the probe power at the input port outside the refrigerator. The saturation effect is highly nonlinear so that we can calibrate the absolute internal photon number with respect to the applied MW power. The strength of the self-Kerr nonlinearity and the attenuation in the input lines are determined from the fits as $\alpha_0/2\pi = -13.0$ MHz and -57.3 dB, respectively.

SUPPLEMENTARY NOTE 6: STARK SHIFT BY THE SAW EXCITATION

To calibrate the SAW input power, we measure the Stark shift of the MW resonator induced by the SAW excitation. Supplementary Figure 5 shows the Stark shift as a function of the phonon number in the SAW resonator at zero flux

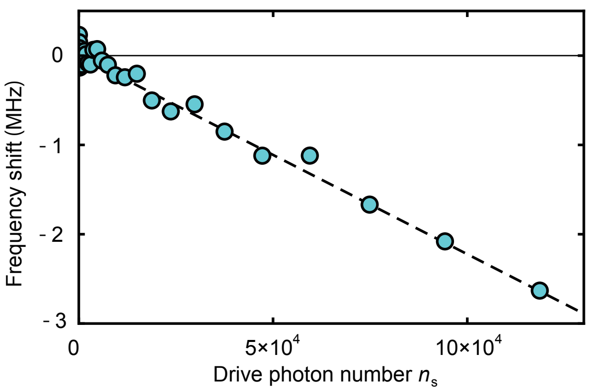

Supplementary Figure 5: Stark shift of the MW resonator due to the excitation of phonons in the SAW resonator.

bias. Here we use the Stark shift per single phonon which is calculated to be

$$\chi_{\mathrm{s}} = \frac{2g_{\mathrm{p}}^2\alpha_0}{\delta^2} = 2\pi \times 22 \text{ Hz}. \tag{S20}$$

The intra-resonator phonon number of the SAW resonator is given as $n_{\mathrm{s}} = 4A_{\mathrm{s}}P_{\mathrm{s}}/\hbar\omega_{\mathrm{s}}\Gamma$, where P_{s} is the drive power at the SAW input port outside the refrigerator. From comparison with the experimental result, the attenuation A_{s} along the SAW input line is determined to be -73 dB including the effect of the external coupling efficiency of the SAW resonator.

* u-atsushi@g.ecc.u-tokyo.ac.jp
