# Supplementary material for: Single-photon quantum regime of artificial radiation pressure on a surface acoustic wave resonator
Source: Nat Commun. 2020 Mar 17;11:1183. doi: 10.1038/s41467-020-14910-z (PMC7078202; doi:10.1038/s41467-020-14910-z)
Supplement: Supplementary file 2 — Peer Review File [file 41467_2020_14910_MOESM2_ESM.pdf]

Editorial Note: This manuscript has been previously reviewed at another journal that is not operating a transparent peer review scheme. This document only contains reviewer comments and rebuttal letters for versions considered at Nature Communications.

REVIEWERS' COMMENTS:

Reviewer #3 (Remarks to the Author):

I read through the revised manuscript. The authors sufficiently addressed all points raised in the previous referee reports. In comparison to the version of the manuscript I have seen previously, the presentation and clarity improved significantly. I recomment the present manuscript for publication at Nature Communications.
